# The Impact of the Green Finance Reform and Innovation Pilot Zone on the Green Innovation—Evidence from China

**DOI:** 10.3390/ijerph19127330

**Published:** 2022-06-15

**Authors:** Yanbo Zhang, Xiang Li

**Affiliations:** School of Business Administration, Northeastern University, Shenyang 110169, China; ybzhang@mail.neu.edu.cn

**Keywords:** green finance, green innovation, difference-in-difference model, panel data

## Abstract

This article uses the “Green Finance Reform and Innovation Pilot Zone” promulgated in 2017 as an example to construct a quasi-natural experiment and uses the difference-in-difference method to test the impact of the implementation of the “Green Finance Reform and Innovation Pilot Zone” on the green innovation activities. It was found that the policy promotes the quantity and quality of corporate green innovation. The mechanism test showed that policy promotes the R&D investment and expands the credit scale. The study further found that green finance policies enhance the green innovation of enterprises as government environmental regulation is strengthened. Finally, green innovation by state-owned enterprises is more strongly promoted in the pilot green finance reform and innovation zones, and green innovation by enterprises in non-polluting sectors is more sensitive to the policy, with a heterogeneous pattern of policy effects in eastern and non-eastern China. Therefore, green finance policies should be promoted to achieve an effective combination of financial resource allocation and corporate green innovation to promote the construction of ecological civilization.

## 1. Introduction

In 2020, China set the goal of achieving “peak carbon” by 2030 and “carbon neutrality” by 2060. As the world’s largest carbon emitter, China has only 30 years to go from “peak carbon” to “carbon neutral”, far below the average level of 70 to 80 years in developed countries. However, achieving the goal of “carbon peaking” and “carbon neutrality” is a huge challenge, with a tight schedule, a heavy task and great difficulty. Equally, this means that economic and social transformation to green and low-carbon is required to achieve win–win and sustainable development for the economy, energy, environment and climate. The transformation of the real economy will face greater pressure. Scientific and technological innovation is the basis and key to supporting China’s goal of carbon neutrality, green innovation is the cornerstone of green development and green transformation of society, and financial bottlenecks are a major impediment to the development of green innovation; insufficient green innovation leads to high costs for green products and services, making the development of green production and green consumption limited. Therefore, in 2016, the People’s Bank of China and seven other departments issued the “Guidance on Building a Green Financial System”, making China the first country in the world to have its central government promote the building of a green financial system. In 2017, the State Council decided to set up “green financial reform and innovation pilot zones” in five pilot provinces across the country, marking a new stage of development for green finance in China, combining top-down design and bottom-up regional exploration.

Enterprises are the mainstay of innovation and play a vital role in China’s efforts to build a world power in science and technology. In the process of corporate innovation, the allocation of financial resources plays a key role, and the increased flow of financial resources to green industries leads to the optimal allocation of resources such as land and labor. In theory, the green finance pilot policy influences corporate innovation through the following channels: firstly, the “debt financing mechanism”—where the green finance pilot policy emphasizes measures such as providing loans at low-interest rates, facilitating bond issuance and listing or providing more favorable premiums to green industries, while adopting punitive high-interest rate loans to polluting industries or refusing to issue loans—prevent enterprises from going public and charge higher premiums and other measures, which largely promote the innovative development of green enterprises and force polluting enterprises to transform and upgrade through innovation. Secondly, for the “innovation input mechanism”, green finance may bring certain “innovation compensation effect” to enterprises, prompting them to increase innovation input to promote enterprise development and eventually form excess returns, which is consistent with the “Porter hypothesis”. Unfortunately, the existing studies have not yet comprehensively tested the above theoretical analysis, and few scholars have explored the impact effects of green finance pilot policies and their mechanisms based on both quantitative and qualitative perspectives of green innovation.

Therefore, how exactly do China’s green finance pilot policies affect corporate green innovation? More importantly, do they significantly increase R&D expenditure, alleviate firms’ financing constraints and promote their innovative development? Are there significant differences in the impact effects of green finance pilot policies between polluting and green sectors? This requires rigorous empirical testing. In view of this, this paper took the pilot green finance policy as an entry point to explore in-depth the implementation effect of the pilot green finance policy and its impact difference and mechanism of action between polluting and green industries from the perspective of micro enterprises, which is of great theoretical and practical significance for deepening the innovative pilot green finance policy, expanding the pilot green finance reform pilot zone and promoting the green and efficient development of enterprises.

## 2. Literature Review

There are two main strands of the literature that are closely related to the research content of this paper. One is on the evaluation of the effects of green financial policies, and the other examines the impact of environmental policies on corporate green innovation.

Regarding the evaluation of the effects of green finance policies, scholars usually discuss the micro impacts of green finance on financial institutions and enterprises. Green finance mainly includes green credit, green bonds, green equity indices and a variety of green financial instruments or services, including related products, green development funds, green insurance and carbon finance. Most of the current domestic and international assessments of the effects of green finance policies focus on green credit and green bonds [1]. On the one hand, the improvement of the quality of development of financial institutions, i.e., the improvement of their profitability and risk management, promotes the development of green credit, which not only better manages the risk of financial institutions but also improves their reputation, overall competitiveness and financial performance, which is achieved by reducing the risk level of commercial banks, improving their risk control capacity and increasing their return on assets. Therefore, financial institutions can choose to support technological innovation within the industry without changing the direction of investment in the lending sector, helping enterprises to get out of difficulties through more sophisticated post-loan management while improving the cleanliness of the industry [2]. On the other hand, green credit has a richer micro impact on enterprises. Green credit policies promote total factor productivity through financial mismatch and debt financing, and green credit policies can also promote green innovation in enterprises and motivate enterprises to focus on front-end prevention and control rather than end-of-pipe emission reduction. Green credit focuses on the invisible environmental impacts of financial operations, and the over-allocation of credit resources to the polluting sector can lead to capital mismatches that can undermine the optimal path of economic growth [3]. The impact of green credit policies on polluting and non-polluting firms can therefore differ. Some scholars found that green credit policies have significant investment disincentive and financing penalty effects, effectively inhibiting the investment behavior of heavily polluting firms, increasing the debt financing costs and debt financing maturity of polluting firms but reducing the debt financing costs of green firms. At the same time, green credit policies significantly exacerbate the exit risk of highly polluting enterprises, with positive market selection effects and market share reallocation effects [4,5,6,7].

The core function of green finance is to promote the optimal allocation of financial resources between the environmental and economic sectors and is essentially an important extension and innovation of traditional environmental policy. Scholars conducted a great deal of research on environmental policy and corporate innovation, and three main views emerged. One is to follow the cost effect. That is, strict environmental governance policies increase the cost of pollution emissions and treatment for enterprises, thus crowding out productive investment and innovation R&D input, making green innovation capacity decline. The second is the innovation compensation effect [8,9,10]. “The Porter hypothesis suggests that strict and reasonable environmental governance policies can stimulate firms to innovate and obtain compensation for product innovation, i.e., the institutional pressure generated by a moderate intensity of environmental regulation promotes firms to increase green innovation-related R&D investment and promote green innovation activities. Third, there is a non-linear relationship between the two. Depending on the form of environmental regulation, there is a statistically significant “U” shaped relationship between the intensity of environmental regulation and firms’ innovation behavior [11]. Environmental regulation pushes firms to increase their R&D intensity and thus promote green technology innovation, but the promotion of green technology innovation has a threshold effect that changes from weak to strong, from insignificant to significant [12,13].

Traditional green finance policy-related research generally revolves around single financial instruments or related policies such as green credit and green bonds, such as the Green Credit Guidelines and the Green Bond Issuance Guidelines. On the whole, green finance is a financial innovation to solve the financing problems of environmental protection industries and projects and is a credit allocation based on environmental constraints. Green finance can establish a green investment and financing incentive mechanism, internalize environmental pollution into the financing costs of emission enterprises, prompt the flow of capital from highly polluting industries to low-polluting industries, reduce the return on investment and availability of capital in polluting industries and increase the Green Finance Reform and Innovation Pilot Zone, a pilot project for the reform and innovation of green finance, which increases the financial support for environmental protection enterprises to achieve green economic development. The Green Finance Reform and Innovation Pilot Zone is a more comprehensive green finance policy, marking the implementation of green finance, and there has been relatively little evaluation and research into this more comprehensive green finance policy. In addition, the existing literature on the impact and mechanism of green finance on the quality and quantity of green innovation of enterprises is relatively limited. Therefore, this paper used the Green Finance Reform and Innovation Pilot Zone policy as a quasi-natural experiment to investigate whether the Green Finance Reform and Innovation Pilot Zone achieved a “quantitative” and “qualitative” increase in corporate green innovation. First, this paper assessed the policy effects of the pilot green finance zone based on the dual dimensions of the quantity of corporate green innovation and the quality of corporate green innovation, which helps to understand the micro policy effects of the pilot green finance policy and is of great significance in promoting corporate green transformation. Second, for the first time, an innovative environmental policy tool, the central environmental protection inspector, was introduced into the study of green finance to analyze whether environmental policy tools can strengthen the positive impact of the green finance reform and innovation pilot zone on enterprises’ green innovation behavior, with a view to enriching the study of environmental policy and enterprises’ green innovation. Thirdly, this paper attempted to reveal the mechanism of green innovation behavior of enterprises in green financial reform and innovation pilot zones and theoretically explored whether green financial reform and innovation pilot zones promote enterprises to increase relevant R&D investment and bring into play the innovation compensation effect; at the same time, whether the policy-induced credit scale expansion channel also affects green innovation of technology enterprises. Fourthly, this paper identified the heterogeneity of policy implementation effects from the perspective of test zone categories and enterprise characteristics. It can provide a reference for the government to formulate and dynamically adjust green credit policies.

## 3. Policy Background and Theoretical Hypothesis

With the accelerated pace of ecological civilization construction and sustainable economic development, green finance is not only a new trend in financial development but also an important way to achieve the carbon neutrality target. At present, China’s financial support for green innovation is seriously inadequate relative to the carbon neutrality target, and it has become a global consensus that green finance can help green innovation and achieve the strategic goal of carbon peaking and carbon neutrality. On 14 June 2017, China selected some places in five provinces (autonomous regions), namely Zhejiang, Jiangxi, Guangdong, Guizhou and Xinjiang province, to build green financial reform and innovation pilot zones with their own focus and characteristics, to explore institutions and mechanisms The pilot zones are designed to explore institutions and mechanisms that can serve as a model and promote the experience. By taking into account the differences in industrial structure, environmental carrying capacity and regional resource endowments, the pilot zones are broadly divided into three categories: the first category is the pilot zones in Guangdong and Zhejiang, where the financial system is more developed, focusing mainly on the development of green financial markets and the exploration of green financial policies and services. The second category is the Jiangxi and Guizhou pilot areas, which are at a slightly weaker level of economic development, focusing on exploring ways to avoid “pollution before treatment”, gradually building up green financial mechanisms and exploring green development models through the application of high-quality green resources. The third category is the Xinjiang pilot zone, which focuses on exploring green finance to help modernize agriculture and clean energy resources according to their industrial structure and resource endowment. The governments of five provinces (autonomous regions), namely Zhejiang, Jiangxi, Guangdong, Guizhou and Xinjiang, issued several special policy documents on green finance, providing theoretical platforms and concrete implementation plans for green finance development through monetary and financial policies, fiscal and taxation policies and a range of other policies.

According to the microeconomic theory of banking, moral hazard and adverse selection arising from information asymmetry between banks and firms are the root causes of credit rationing [11]. With limited endogenous financing and China’s predominantly indirect financial structure, bank credit has become an important source of funding for enterprises’ innovation activities. The Green Finance Reform and Innovation Experimental Zone influenced enterprises’ green innovation behavior by changing the sectoral flow of financial resources, supporting enterprises to carry out clean technology transformation and encouraging financial institutions to develop diversified green financial products. The willingness of enterprises to behave in line with policy standards under the pressure of green finance policies helps build a good corporate social image, making it easier for them to gain the trust of investors. Firms also have access to larger-scale and longer-term external financing [14,15]. This reduces the uncertainty of their future operations and increases the stability of their cash flows, which in turn increases their resilience and solvency to green innovation. Compared to other innovation activities, green innovation is not only characterized by high investment, high risk and a long lead time but also has strong environmental externalities, which makes it necessary for enterprises to have long-term stable financial support for green innovation activities and to avoid financial risks caused by short-term debt pressure as far as possible, in order to provide long-term financial security for their innovation activities. As “rational economic agents”, companies have a strong incentive to innovate green, both in terms of access to finance and for the purpose of optimizing their social image [16].

Corporate R&D investment can significantly enhance the output of innovation. The higher the intensity of R&D investment, the more patent output a firm obtains. When faced with environmental and economic policies, enterprises generally have two strategic choices: one is to accept the environmental costs brought about by the policy and directly treat pollution to meet the policy requirements, i.e., the cost-shifting strategy [15,17,18]; the other is to increase R&D investment to meet the environmental policy requirements while gaining efficiency gains from innovation, i.e., the innovation R&D strategy [19,20]. Following the launch of the Green Finance Reform and Innovation Pilot Policy, banks in the pilot areas included corporate environmental performance as a mandatory condition for credit approval. When the external pressure generated by environmental performance threatens a company’s source of funding, the company has huge market demand for environmental technology, which stimulates the impulse to innovate and increase technological innovation [11]. According to the Porter hypothesis effect, when the compensation effect of the triggered innovation can offset or even exceed the cost of regulation, the productivity of enterprises will also increase. Therefore, the Green Finance Reform and Innovation Experimental Zone can stimulate enterprises to increase their R&D investment, which in turn increases their green innovation output and create an “innovation compensation” effect.

In summary, the establishment of the Green Finance Reform and Innovation Experimental Zone promotes the increase in R&D investment and credit expansion of enterprises in the pilot areas, which, in turn, promotes green innovation. Accordingly, this paper proposes the theoretical hypothesis H1.

**Hypothesis** **1** **(H1).** *The establishment of a green financial reform and innovation experimental zone will promote the increase in R&D investment and credit expansion of enterprises in the pilot area, which will, in turn, promote green innovation of enterprises*.

The effectiveness of green finance policies requires the support of environmental protection policies and related laws and regulations. The strengthening of environmental regulations can effectively improve the efficiency of resource allocation for green finance. For example, improving environmental enforcement and strengthening environmental protection interviews can prompt local enterprises to reduce pollution emissions and increase green innovation, and strict environmental regulation has shown good results in terms of the actual effects of pollution prevention and control. The Central Environmental Protection Inspectorate is a new environmental regulation policy in recent years, which provides effective supervision of the process of environmental enforcement. There is no doubt that the Central Environmental Protection Inspectorate has been a catalyst for green innovation in enterprises. At the same time, environmental regulation can encourage innovation and technological progress, which in turn can improve the efficiency of production management and the competitiveness of enterprises, offsetting the rising costs of environmental regulation. The strengthening of environmental regulations can optimize the allocation of financial resources and direct the flow of capital to green industries, thus promoting corporate green innovation. Therefore, this paper proposes research hypothesis H2.

**Hypothesis** **2** **(H2).** *Central environmental protection inspectors can strengthen the promotion effect of green financial pilot policies on enterprises’ green innovation.*

Regional resource endowments and the degree of financial development also affect the concrete implementation of green finance policies. On the one hand, green financial policies encourage green innovation by enterprises. Provinces with developed economies and resource endowments have more developed innovation systems, as well as more advantageous human, material and financial resources, which are conducive to corporate green innovation. On the other hand, green financial policies guide the allocation of funds for corporate loans. When the financial system of a region is more developed, the access to financing for enterprises is more optimized. In regions with a well-developed financial sector, the funding orientation of green finance policies is enhanced. In regions with less developed financial development, on the other hand, enterprises face a tougher financing environment and limited financing options. Therefore, the innovation incentives of green credit policies should be weaker for enterprises in regions with less developed financial development. Based on the above analysis, this paper proposes research hypothesis H3:

**Hypothesis** **3** **(H3).** *After the establishment of the green financial reform and innovation experimental zone, the green innovation of enterprises in developed provinces will be more significantly increased compared to less-developed provinces*.

The nature of ownership of enterprises differs, and the marginal costs they can bear also differ, so enterprises need to weigh the sources of company funds and the allocation of corporate funds in the process of operation. Currently, China’s financial resources are captured by state-owned enterprises, and when green finance policies are piloted, non-state-owned enterprises lack incentives to innovate due to their inability to maintain a high level of sensitivity to policy implementation. In contrast, prior to the implementation of the policy, the sources of funding involved in SOEs are usually greater, leading to a greater impact of the green finance policy on them and sufficient funds for innovation. Therefore, SOEs should be more motivated to innovate, improve their own green development and respond to the green finance policy dividend. Based on the above analysis, this paper proposes the following research hypothesis H4:

**Hypothesis** **4** **(H4).** *After the establishment of the Green Finance Reform and Innovation Experimental Zone, the green innovation of state-owned enterprises will increase more significantly compared to non-state-owned enterprises*.

The implementation of green finance policies will make it more difficult for enterprises in polluting industries to raise funds, and the available sources of funds will decline, and fewer funds will be available for R&D activities. From the bank’s perspective, the green finance policy exposes banks to various risks when granting loans: firstly, default risk, as the negative environmental externalities caused by polluting enterprises or the strengthening of environmental regulations cause the production costs of polluting enterprises to rise and the profits of enterprises to fall or even close down, banks may face the risk of not being able to recover their loans; secondly, reputation risk, as financial institutions granting loans to enterprises in polluting industries are not in line with policy guidelines Third, the risk of declining returns. As profitable financial institutions, banks tend to provide more financial resources to non-polluting industries in order to obtain more robust returns under the principle of profit maximization. Based on the above analysis, this paper proposes research hypothesis H5:

**Hypothesis** **5** **(H5).** *After the establishment of the Green Finance Reform and Innovation Experimental Zone, the green innovation of enterprises in non-polluting industries will increase more significantly compared to polluting industries.*

## 4. Data and Methods

### 4.1. Sample Selection and Data Sources

In this paper, the initial sample of Chinese A-share listed industrial enterprises was selected for the period 2010–2019 and treated with reference to the previous literature as follows: (1) in order to avoid the influence of outliers, enterprises with outliers in their financial status, such as ST, *ST and PT, were removed from the original data; (2) enterprises with severely missing data on the main variables were excluded, and the missing values of individual variables were interpolated; (3) in order to eliminate the influence of extreme values, the main continuous variables were trimmed at the 1% level. The data on corporate green innovation were obtained from the Incopat patent database, other corporate-level data were mainly obtained from the Guotai Junan database and the Wind database and were collated and supplemented with company annual reports, while other sample data were mainly obtained from the China Statistical Yearbook, etc. After a series of screening and processing, unbalanced panel data of 14,779 observations were obtained.

### 4.2. Definition of Variables

(1) Explanatory variable: Corporate green innovation ($Y_{it}$), this paper refers to Wang’s study [5].

The total number of patent applications (including inventions, utility models and designs) (Greenpat) was used as a basic indicator of the “quantity” of green innovation, while green invention patents have the highest degree of innovation compared to green utility models and green design patents. The number of green invention patent applications (Greenino) is used as a basic indicator of the “quality” of green innovation. In order to eliminate the problem of the right-skewed distribution of green patent applications, the number of green patent applications was added by one, and the natural logarithm was taken to obtain Lnpat and Lnino;

(2) Explanatory variable: The Green Finance Reform and Innovation Experimental Zone (Gfinance). The use of quasi-natural experiments to examine the effects of policies is a common method in recent years; green financial reform and innovation pilot zones as exogenous shocks provide the opportunity for quasi-natural experiments using the difference-in-difference method, i.e., whether or not to be established as a pilot zone is not influenced by individual choices such as province (city). The interaction term between the innovation zone and the time of establishment was chosen here as the explanatory variable;

(3) Control variables: The following firm-level and province-level control variables were introduced to synthesize the existing literature.

The main firm-level control variables affecting total factor productivity are: gearing (Lev), expressed as the ratio of total liabilities to total assets; return on net assets (Roa), expressed as the ratio of profit after tax to net assets; firm size (Size), expressed as the natural logarithm of total assets; Tobin’s Q, expressed as the ratio of the firm’s market value to the replacement cost of its assets; equity cash flow from operating activities (Cash), expressed as the ratio of net cash flow from operating activities to total assets; age of the firm (Age), expressed as the age of the firm as of the current period (i.e., statistical year—firm establishment + 1); the control variables at the provincial level that affect green innovation in enterprises are the level of economic development and the total population of the city, both logarithmically. The specific descriptive statistics of the variables are shown in Table 1 and Table 2.

From the descriptive statistics of the main variables in this paper, the mean values of the quantity of green innovation and the quality of green innovation of enterprises are 3.950 and 0.764, which shows that the total number of green invention patents is relatively small compared to the total number of green patent applications, and the other control variables are basically consistent with the established studies.

### 4.3. Methods

The differences in green innovation among firms after the establishment of the Pilot Green Finance Reform Zone arise from three sources: the time effect, the attribute difference and the policy effect. The time effect refers to the characteristic that green innovation still changes over time for firms in non-trial green financial reform zones; the attribute difference refers to the fact that the characteristics of different cities or firms affect green innovation; the policy effect refers to the change in green innovation of firms caused by the establishment of the pilot green financial reform zones. The difference-in-difference method measures the net effect of the policy treatment by identifying differences in both the between-group and time dimensions. Considering that the establishment of the pilot green financial reform zone has a policy radiation effect on the provinces where it is located, listed companies in the five provinces of the pilot zone are used as the experimental group, and listed companies in other provinces are used as the control group. A benchmark regression model was constructed as follows [21,22].

(1) $$Y_{it}=\beta_{0}+\beta_{1}Gfinance_{jt}+\beta_{2}X_{it}+v_{i}+\gamma_{t}+e_{it}$$

where *i*, *j* and *t* denote firm, province and time, respectively. The difference-in-difference term $Gfinance=treat_{i}*time_{t}$ is the core explanatory variable. $treat_{i}$ is a dummy variable for the pilot region, taking 1 for enterprises in the five provinces (regions) of Zhejiang, Jiangxi, Guangdong, Guizhou and Xinjiang, and 0 for enterprises in the remaining provinces. $time_{t}$ is a dummy variable for the period before and after the policy pilot, with 1 taken during the policy implementation period (2017 and after) and 0 taken before the policy implementation (2017 and before).

$Y_{it}$ is the explanatory variable; specifically the total number of green patent applications (Lnpa) and the number of green invention patent applications (Lnino). $X_{it}$ is a set of control variables, including firm-level gearing, return on net assets, firm size, Tobin’s Q, equity concentration, cash flow from operating activities, firm age and province-level GDP and population per capita. $v_{i}$ is individual fixed effects, $\gamma_{t}$ is the time fixed effects, and $e_{it}$ are random error terms. For the coefficients in the model $\beta_{1}$, the magnitude and sign of the coefficients directly reflect the net effect of the environmental talks, and if the Green Finance Reform Pilot Zone has a catalytic effect on corporate green innovation, then $\beta_{1}$ should be significantly positive.

## 5. Results and Analysis

### 5.1. Baseline Regression Results

Based on model (1), Table 3 reports the regression results of the Green Finance Reform and Innovation Pilot Zone on corporate green innovation. Column (1) reports the regression results for total green innovation. The coefficient of the cross-product term is significantly positive at the 1% level, with a coefficient of 0.3446. i.e., the total number of green patent applications in the pilot provinces (regions) increased by about 34% after the establishment of the pilot zone for green financial reform and innovation, indicating that the pilot zone for green financial reform and innovation significantly increased the number of green innovations by enterprises in the pilot zone. Column (2) reports the results of the regression of green invention patents. The coefficient of the cross-product term is significantly positive at the 1% level, with a coefficient of 0.1116, i.e., the total number of green patent applications in the pilot provinces (districts) increased by about 11% after the establishment of the pilot zone for green financial reform and innovation, indicating that the pilot zone for green financial reform and innovation significantly improved the quality of green innovation of enterprises in the pilot zone. This regression result indicates that the Pilot Zone for Green Financial Reform and Innovation has significantly contributed to the improvement of both the quantity and quality of green innovation of enterprises in the Pilot Zone.

### 5.2. Robustness Tests

In order to ensure the robustness of the study results, this section focuses on robustness testing using the replacement of explanatory variables, parallel trend tests and counterfactual tests.

(1) Replacing the explanatory variables. Column (3) of Table 2 shows the regression results of the replacement of the explanatory variables. Although the number of green patent applications is the most widely used indicator to measure the innovation performance of enterprises, it has the disadvantage of not fully reflecting the innovation projects of enterprises, and new product sales revenue is chosen here as a proxy variable for green innovation of enterprises for robustness testing. New product sales revenue is an indicator of a company’s innovation achievements, i.e., the success of new products brought to market, and is used to reflect the market value of a company’s green innovation. The logarithm of new product sales revenue (Lnnproduct) is regressed separately as an explanatory variable. Column (3) reports the regression results, with the coefficient of the cross product term being significantly positive at the 1% level, with a coefficient of 0.1250. This regression result is consistent with the underlying regression results, and the underlying regression results are robust.

(2) Parallel trend test. The use of the DID method requires the parallel trend test to be satisfied, i.e., the trend of total factor productivity changes in the control group and the experimental group should remain roughly the same in the absence of external shocks [23]. In order to test whether the total factor productivity of the treatment and control groups satisfies the parallel trend hypothesis, the following regression model was set up on the basis of model (1).

(2) $$Y_{it}=\beta_{0}+{\sum_{b}D}_{b}Before_{jb}+B_{1}Currer_{jt}+{\sum_{a}A}_{a}After_{ja}+\alpha X_{it}+v_{i}+\gamma_{t}+e_{it}$$

where *b* indicates the impact in period b before the treatment, *a* indicates the impact in period a after the treatment, and the coefficient $\beta_{1}$ indicates the impact of the treatment period, i.e., the dummy variable takes the value of 1 when a year is the treatment period; otherwise, it takes the value of 0. Other symbols have the same meaning as in model (1).

Table 4 reports the results of the test, where there was no significant difference in the trend of change in the dependent variable between the experimental and control groups in the six periods prior to the pilot green financial reform and innovation zone. The following conclusions were obtained: the hypothesis of parallel trends holds, and the number of green innovations and the quality of green innovations of the sample firms in the experimental and control groups had the same trend of change before the pilot.

(3) Counterfactual test. During the sample period, some policies or unobservable influences other than the Green Finance Reform and Innovation Pilot Policy also led to changes in corporate green innovation, so changes in green innovation may not be related to the Green Finance Pilot Policy. In order to test the reasons for the change in enterprises’ green innovation during the sample period, a counterfactual test was conducted by changing the timing of the policy pilot, i.e., regressing the pilot policy of the green financial reform and innovation pilot for two years earlier as a time dummy variable, and if the regression coefficient of the green financial pilot policy on enterprises’ green innovation is still significantly positive at this time, it indicates that the increase in the quantity (quality) of enterprises’ green innovation in the pilot area may be due to the influence of other unobservable factors or policies, not all of which are caused by the green finance pilot policy, as shown in columns (1) and (2) of Table 5. The estimated coefficients of the difference-in-difference term are not significant after the policy was implemented two years earlier, which indicates that the result that the policy of the pilot green finance reform and innovation zone promotes enterprises’ green innovation is robust during the sample period.

## 6. Mechanism of Action Test

### 6.1. Intermediary Effects Test

According to the previous analysis, it can be found that the green financial reform and innovation pilot zone policy promoted the quantity and quality of green innovation in enterprises. This paper further examined the mechanism by which the Green Finance Reform and Innovation Pilot Zone policy promoted corporate green innovation based on the baseline analysis.

As explained in the theoretical analysis, the pilot policy is likely to promote green innovation through increased investment in R&D and credit expansion by firms in the pilot areas.

Drawing on the study by Wen Zhonglin et al. and Chen Renjun et al. (2019), a mediating effect model was used to explore the mechanism of action, with the ratio of corporate R&D expenditure to operating revenue as a proxy variable for R&D investment (R&D) and the ratio of long-term corporate borrowing to total assets as a proxy variable for credit size (Debt). The mediating effects model was constructed as follows.

(3) $$Y_{it}=\beta_{0}+\beta_{1}Gfinance_{jt}+\alpha X_{it}+\nu_{i}+\gamma_{t}+e_{it}\;$$

(4) $$Media_{it}=\delta_{0}+\delta_{1}Gfinance_{jt}+\sigma X_{it}+\nu_{i}+\gamma_{t}+e_{it}\;$$

(5) $$Y_{it}=\theta_{0}+\theta_{1}Media_{it}+\theta_{2}Gfinance_{it}+\omega X_{it}+\nu_{i}+\gamma_{t}+e_{it}$$

If there is a significant positive effect of the pilot policy on both credit size and R&D investment, the regression coefficients $\beta_{1}$ and $\delta_{1}$ should be significantly positive. After introducing the mediating variables in model (4), the regression coefficients of the pilot policy on firms’ green innovation $\theta_{2}$ is still significant, but compared to $\beta_{1}$, there is a decrease, indicating that R&D costs and credit size are some of the mediating variables. Table 6 reports the estimated results of the mediating effects: the increase in R&D investment and credit scale expansion of enterprises are partial mediating variables that promote the quantity and quality of green innovation of enterprises, i.e., the green financial reform and innovation pilot zone policy can help enterprises’ green innovation by promoting the increase in R&D investment and credit scale expansion.

Table 6 reports the regression results of the mediating effect of R&D investment: firms’ R&D investment is part of the mediating variable between the green finance pilot policy and firms’ green innovation quality, and the regression coefficients in columns (2) and (3) are both significant at the 1% confidence level, thus indicating that increased R&D investment is a channel through which the green finance pilot policy promotes the quantity and quality of listed green innovation.

Table 7 reports the results of the mediating effect of credit size: the credit size of enterprises is part of the mediating variable between the green finance pilot policy and the quantity of green innovation of enterprises. The regression coefficient in column (2) is significant at a 1% confidence level, thus indicating that credit size expansion is the channel through which the green finance pilot policy promotes the quantity of listed green innovation. The regression coefficient in column (3) is insignificant, indicating that there is no channel of credit scale expansion in the mechanism of the green finance pilot policy to promote the quality of green innovation of enterprises.

### 6.2. Central Environmental Inspection and the Testing of Green Finance Pilot Policies

The strengthening of environmental regulations can effectively improve the efficiency of resource allocation for green finance. Improving environmental enforcement and strengthening environmental interviews can prompt local enterprises to reduce pollution emissions and increase green innovation. In terms of the actual effect of pollution prevention and control, strict environmental regulations have shown good results. The green finance pilot policy has driven social capital into the eco-industry, and the Central Environmental Protection Inspectorate (CEPI) policy in 2017 has increased the intensity of environmental regulation; can the CEPI strengthen the green finance pilot to improve the quantity and quality of green innovation of enterprises? In order to explore this question, model (5) was constructed as follows.

(6) $$Y_{it}=\rho_{0}+\rho_{1}EG_{3}{}_{jt}+\rho_{2}X_{it}+v_{i}+\gamma_{t}+e_{it}$$

where the difference-in-difference term $EG_{3}=Evironment_{3}*Gfinance_{3}$. If a province in the t year is both a central environmental inspection and a green finance pilot area, then $Er_{3}=1$, otherwise $Er_{3}=0$ is 0. $\theta_{1}$ is the coefficient of the cross term, reflecting the impact of the double-difference between the green finance pilot policy and the central environmental protection inspection on the development of green innovation of enterprises. If the central environmental protection inspection can strengthen the pilot policy to enhance the quantity and quality of enterprises’ green innovation, then the regression coefficient $\rho_{1}$ should be significantly positive and larger than the coefficient in model (1). For $\beta_{1}$, the regression coefficients should be significantly positive and larger than those in model (1). Table 8 reports the regression results: the regression results in column (1) indicate that the central environmental protection inspection strengthens the positive effect of the pilot green finance policy on the quantity of green innovation of enterprises, while the regression results in column (2) show that there is no evidence that the central environmental protection inspection strengthens the positive effect of the pilot green finance policy on the quality of green innovation of enterprises. This may be caused by the longer time required for firms to patent their green inventions and the weaker persistence of the central environmental protection inspection policy.

## 7. Heterogeneity Analysis

In order to further explore the heterogeneity of green finance pilot policies, this paper conducts a heterogeneity analysis based on the characteristics of the existing sample, such as the type of pilot zone, enterprise ownership and enterprise industry, in order to distinguish which enterprises are more significantly affected by green finance pilot policies.

### 7.1. Test for Heterogeneity of Test Area Categories

The pilot zones can be divided into three categories according to their functions: the first category is the pilot zones in Guangdong and Zhejiang, where the financial system is more developed, mainly focusing on the development of green financial markets and the exploration of green financial policy services. The second category is the pilot zones in Jiangxi and Guizhou, which are at a slightly weaker level of economic development, gradually building up green financial institutions and mechanisms and exploring green development models through the application of high-quality green resources. The third category is the Xinjiang pilot area, which focuses on exploring green finance to help modern agriculture and clean energy resources. After taking into account the differences in resource endowments of each region, the regions to which the sample enterprises belonged were divided into eastern regions and central and western regions for sub-sample regression to examine the differences in policy effects between the eastern regions with more developed financial systems and the central and western regions. The results of the test area category heterogeneity analysis are shown in Table 9. After controlling for other influencing factors, the green finance pilot policy significantly contributed to the growth in the number of green innovations by enterprises in the eastern region and had no significant effect on the number of green innovations by enterprises in the central and western regions. The green finance pilot policy significantly contributed to the growth in the quality of green finance in both the eastern region and the central and western regions. The pilot green finance policy has a better effect on the quality of green innovation of enterprises in the eastern region than those in the western region.

Firstly, a perfect and systematic innovation environment is more conducive to promoting enterprises to increase their investment in research and development, making it easier for them to improve their innovation efforts and thus effectively promote the quality and quantity of their innovation. Moreover, the financial system in the eastern region is more developed, making it easier to obtain long-term loans, which is conducive to corporate innovation.

### 7.2. Test for Heterogeneity of Business Ownership

In this paper, the total sample was divided into state-owned enterprises and non-state-owned enterprises, and the regression estimation of equation (1) was carried out; the specific estimation results are shown in Table 10. As can be seen from Table 10, the coefficients of the interaction terms in columns (1), (2) and (3) are significantly positive, while the coefficient of the interaction term in column (4) is not significant. The coefficients of the interaction terms of the SOE sample are larger than those of the non-SOE sample. This indicates that after the introduction of the green finance pilot policy, the quality and quantity of green innovation of SOEs increased more than that of non-SOEs, while the quality of green innovation of non-SOEs did not improve. In other words, compared to non-SOEs, SOEs are more vulnerable to policy shocks. This is mainly due to the fact that SOEs have obvious policy responsiveness due to their attributes. In addition to pursuing economic interests, SOEs also take on more social and environmental responsibilities and government environmental governance objectives, which increases their incentive to innovate.

### 7.3. Testing for Industry Heterogeneity of Firms

This paper further examined whether the effect of the Green Finance Reform and Innovation Pilot Zone policy on enterprises’ green innovation is affected by the degree of pollution of the enterprises and divided the sample enterprises into two sub-samples: polluting and non-polluting. Specifically, it refers to the study by Li et al. [24]. The heavy polluting industries include 16 industries such as coal, mining, textile, tannery, paper, petrochemical, pharmaceutical, chemical, metallurgy and thermal power, while the control group is industrial enterprises in other industries. The regression results are shown in Table 11. The coefficients of the interaction term of the number of green innovations in the sub-sample of heavy polluters and the sub-sample of non-polluters were both significantly positive. However, the coefficient of the interaction term of the sub-sample of non-polluting enterprises was smaller than that of the interaction term of non-polluting enterprises, indicating that the policy of the pilot green financial reform and innovation zone had a stronger effect on promoting green innovation in non-polluting enterprises compared with that of heavily polluting enterprises. On the one hand, the green financial policy makes the funds that polluting enterprises may use for R&D investment restricted, the green innovation funds of polluting enterprises are limited and the improvement of the green financial system is yet to push further polluting enterprises to increase R&D investment and carry out green innovation. On the other hand, the green financial policy has introduced a series of restrictive policy requirements for the heavily polluting industries, such as gradually reducing the scale and proportion of loans to the “two high and one leftover” industries, discouraging polluting investments and eliminating backward production capacity in traditional industries, etc. As a result, enterprises in heavily polluting industries face stronger financing constraints and greater pressure to survive than non-heavily polluting industries, resulting in less green innovation.

## 8. Conclusions and Policy Recommendations

In order to be able to study the impact of the Green Financial Reform and Innovation Pilot Zone policy on corporate green innovation in-depth, the policy effect was assessed based on the quantitative and qualitative perspectives of green innovation, using data from Chinese A-share listed enterprises from 2010 to 2019, using a difference-in-difference method. The results show that: (i) the green financial reform and innovation pilot zone policy does have a significant promotion effect on both the quality and quantity of enterprises’ green innovation, and other proxies for green innovation also yield consistent findings, indicating that the green financial reform and innovation pilot zone policy achieved significant results, and the implementation and promotion of green finance do have a significant promotion effect on green innovation, providing a basis for the green financial innovation pilot zone experience This result is also supported by parallel trend tests and counterfactual tests, among others. This paper examines the mechanism through which green finance affects corporate green innovation, examining two channels, namely corporate R&D investment and debt size, and finds that green finance does promote the increase in the quantity of corporate green innovation through the channel of increasing the proportion of corporate R&D investment and long-term borrowing, but there is no credit size expansion channel in the improvement of corporate green innovation quality. In addition, this paper introduced the environmental policy of the Central Environmental Protection Inspection and found that environmental regulation can enhance the role of corporate green innovation in the pilot green finance policy. In terms of heterogeneity, there are significant differences in the impact of green finance on the green innovation of enterprises in different types of pilot zones, different ownership and different industries. Specifically, the analysis of heterogeneity by pilot zone category shows that the effect of green finance on green innovation of enterprises in the eastern region is stronger. State-owned enterprises are more sensitive to green finance policies. The pilot green finance policy has a stronger effect on promoting green innovation among non-polluting enterprises than polluting enterprises.

Based on the above findings, this paper makes the following policy recommendations.

Firstly, the pilot zone for green financial reform and innovation should explore replicable experiences and be replicated on a broader scale. The pilot zone for green financial reform and innovation is an important practical exploration of China’s use of financial regulation and other market instruments, and the pilot policy allows each pilot region to build pilot zones that are tailored to local conditions, with their own focus and characteristics, based on the regional institutional environment and resource endowment. Differentiated policies are set according to the type of enterprise, and the policy is revised in a timely manner to optimize the allocation of resources further. It has a certain effect on inducing green innovation in enterprises.

Secondly, a clear guidance program should be formulated for the heavily polluting industries to stimulate enterprises to innovate on their own. For the green innovation of heavily polluting enterprises, the government should further improve the financial support and innovation incentive policies, such as providing government subsidies to enterprises with actual technological innovation needs while strengthening the supervision of their funds to ensure the use of funds in place, forming a number of major projects and pilot demonstration projects with good emission reduction effects and can be replicated; heavy polluting industries are the main target of the green financial reform and innovation pilot zone. The pilot green finance policy should make environmental risks visible, raise the cost of pollution and force enterprises in heavily polluting industries to innovate.

Thirdly, a development model of “government environmental regulation and market capital guidance” should be formed to stimulate more social capital to invest in corporate green innovation. From the perspective of environmental authorities, it is important to ensure the timeliness, effectiveness and authority of environmental law enforcement; increase environmental investigations and punishments in high-environmental and social-risk industries; establish a platform for sharing environmental information on enterprises; and play a positive role in promoting environmental regulation in green financial policies. The government should give full play to the function of environmental regulation, promote the market to guide more social capital into green industries, provide financial support for green innovation and form a new model of green financial reform and innovation.

Compared to Western countries such as Europe and the United States, the green finance policy started late in China. Based on the implementation of the new development concept, the People’s Bank of China has identified green finance as a key task, and in terms of monetary policy, through support tools such as preferential interest rates and special green refinancing, financial institutions are incentivized to provide financial support for carbon emission reduction and better serve the goal of carbon peaking and carbon neutrality, thus accelerating the promotion of financial resources and environmentally optimizing the overall layout of resources. As an important way to achieve the goal of carbon neutrality, how should China’s green finance work? The current incentive mechanism for green financing is not yet perfect. For financial institutions, the return on investment for some green finance projects is low, and some of the social benefits cannot be directly translated into economic benefits. It is recommended that future policy consideration be given to increasing the banking sector’s incentive to support green industries by, for example, reducing the risk weighting of green assets and giving priority to the payment of interests in green assets. At the same time, China should combine its local advantages and start from within enterprises, for example, by adopting incentives such as tax exemptions or tax reductions to encourage enterprises to carry out independent innovation and better play the role of green finance as a guide for capital allocation [25].

## Figures and Tables

**Table 1:** Variable description.

Variable	Statistic	Variable Type
Number of green innovations	Lnpat	Explained variables
Green Innovation Quality	Lnino	Explained variables
Policy	Gfinance	Explanatory variables
Gearing ratio	Lev	Control variables
Return on net assets	Roa	Control variables
Business size	Size	Control variables
Tobin’s Q	Q	Control variables
Concentration of shareholding	Cr	Control variables
Cash flow from operating activities	Cash	Control variables
Age of business	Age	Control variables
GDP per capita	Lgdp	Control variables
Total population	Lpeo	Control variables

**Table 2:** Descriptive statistics.

Variable	Obs	Mean	St. Dev.
Lnpat	14,777	3.950	3.796
Lnino	14,777	0.764	1.082
Gfinance	14,779	0.192	0.394
Lev	14,779	0.412	0.226
Roa	14,779	0.043	0.069
Size	14,779	22.099	1.261
Q	14,764	2.139	1.957
Cr	14,779	34.765	14.695
Cash	14,779	0.061	0.525
Age	14,779	16.39	5.553
Lgdp	14,779	10.952	0.470
Lpeo	14,779	3.984	0.639

**Table 3:** Basic return.

	(1)	(2)	(3)
Variable Name	Lnpa	Lnino	Lnnproduct
Gfinance	0.3446 ***	0.1116 ***	0.1250 ***
	(0.0536)	(0.0171)	(0.0477)
Lev	−0.0882	−0.0170	−0.6697 ***
	(0.1715)	(0.0518)	(0.1922)
Roa	0.2052	−0.1696	−0.4336
	(0.4271)	(0.1301)	(0.4389)
Size	1.9643 **	2.3779 ***	14.3601 ***
	(0.8670)	(0.2545)	(0.8646)
Q	−0.0033	0.0112 ***	0.0197
	(0.0131)	(0.0040)	(0.0143)
Cr	0.0009	−0.0004	0.0090 ***
	(0.0026)	(0.0008)	(0.0026)
Cash	−0.2720 ***	−0.0320	0.0512
	(0.1046)	(0.0320)	(0.1100)
Age	−0.0464	−0.0145	−0.6478
	(0.0391)	(0.0120)	(4.5415)
Lgdp	0.8212 ***	0.2828 ***	−0.2535
	(0.0862)	(0.0253)	(0.2870)
Lpeo	0.5220 ***	0.1026 ***	0.0514
	(0.1107)	(0.0303)	(0.3176)
Constant	−13.0629 ***	−10.0710 ***	−7.0235 ***
	(2.4567)	(0.7275)	(0.8160)
Observations	14,762	14,762	7133
R-squared	0.3872	0.6631	0.3326
year FE	YES	YES	YES
individual FE	YES	YES	YES

Notes: *** and ** denote significance levels of 1% and 5% respectively; values in brackets are robust standard errors of clustering, and “YES” indicates controlling for firm and year fixed effects, as below.

**Table 4:** Parallel trend test.

	(1)	(2)
Variable Name	Lnpa	Lnino
before 6	0.0993	0.1001
	(0.0789)	(0.0995)
before 5	0.4505	0.1382
	(0.3097)	(0.0876)
before 4	0.0959	0.1218
	(0.0761)	(0.0818)
before3	0.4505	0.2257
	(0.3097)	(0.2326)
before 2	0.3340	0.3339
	(0.2790)	(0.2789)
before 1	0.2531	0.2265
	(0.2691)	(0.2807)
Current	0.3225 ***	0.1212 *
	(0.0655)	(0.0728)
after 1	0.3496 ***	0.1813 **
	(0.0935)	(0.0792)
after 2	0.2150 ***	0.1637 **
	(0.0824)	(0.0673)
Constant	48.8976 ***	20.3127 ***
	(2.4132)	(0.6823)
Observations	14,762	14,762
R-squared	0.7641	0.7640
Control variables	YES	YES
year FE	YES	YES
individual FE	YES	YES

Notes: ***, ** and * denote significance levels of 1%, 5% and 10%, respectively; values in brackets are robust standard errors of clustering, and “YES” indicates controlling for firm and year fixed effects, as below.

**Table 5:** Counterfactual test.

	(1)	(2)
Variable Name	Lnpa	Lnino
Gfinance1	0.0302	0.2419
	(0.0517)	(0.1542)
Lev	−0.0253 *	0.3114 ***
	(0.0152)	(0.1057)
Roa	1.7320 *	1.8399 ***
	(0.9355)	(0.2743)
Size	0.4030	−0.0710
	(0.4308)	(0.1305)
Q	−0.2706 ***	−0.0322
	(0.1045)	(0.0317)
Cr	0.1802 ***	−0.0007
	(0.0315)	(0.0046)
Cash	−0.0600	−0.0194
	(0.0392)	(0.0119)
Age	−0.0004	−0.0002
	(0.0026)	(0.0008)
Lgdp	0.5374 ***	0.0953 ***
	(0.1106)	(0.0304)
Lpeo	0.7746 ***	0.1969 ***
	(0.1589)	(0.0447)
Constant	−12.0706 ***	−7.6105 ***
	(3.3974)	(0.9856)
Observations	14,762	14,762
R-squared	0.3728	0.3930
year FE	YES	YES
individual FE	YES	YES

Notes: *** and * denote significance levels of 1% and 10% respectively; values in brackets are robust standard errors of clustering, and “YES” indicates controlling for firm and year fixed effects, as below.

**Table 6:** Tests for mediating effects of R&D investment.

	(1)	(2)	(3)
Variable Name	R&D	Lnpa	Lnino
Gfinance	0.1822 ***	0.2474 ***	0.0381 ***
	(0.0476)	(0.0279)	(0.0057)
R&D		0.1705 ***	0.1620 ***
		(0.0154)	(0.0472)
Lev	0.8547 ***	−1.0065 ***	−0.2675 ***
	(0.1454)	(0.1903)	(0.0539)
Roa	−0.9155	14.8655 ***	6.0359 ***
	(0.5646)	(0.7382)	(0.2090)
Size	−0.5601	1.8700 ***	0.0158
	(0.4637)	(0.6060)	(0.1716)
Q	−0.1158	−0.3004 *	−0.0289
	(0.1369)	(0.1789)	(0.0506)
Cr	0.3463 ***	0.0317	0.2138 ***
	(0.1298)	(0.0203)	(0.0478)
Cash	0.0042	−0.1017 ***	−0.0198 ***
	(0.0046)	(0.0061)	(0.0017)
Age	−0.0024	−0.0031	−0.0018***
	(0.0016)	(0.0021)	(0.0006)
Lgdp	−0.1077 ***	0.6626 ***	0.1386 ***
	(0.0366)	(0.0479)	(0.0136)
Lpeo	−0.0424	0.5881 ***	0.2035 ***
	(0.0594)	(0.0776)	(0.0220)
Constant	3.1965 *	−49.7578 ***	−20.4601 ***
	(1.8378)	(2.4026)	(0.6802)
Observations	14,764	14,762	14,762
R-squared	0.1572	0.6833	0.4393
year FE	YES	YES	YES
individual FE	YES	YES	YES

Notes: *** and * denote significance levels of 1% and 10% respectively; values in brackets are robust standard errors of clustering, and “YES” indicates controlling for firm and year fixed effects, as below.

**Table 7:** Tests of the mediating effect of credit size.

	(1)	(2)	(3)
Variable Name	Debt	Lnpa	Lnino
Gfinance	0.0075 ***	0.1970 ***	0.1623
	(0.0018)	(0.0518)	(0.1287)
Debt		0.0182 ***	0.0192 ***
		(0.0048)	(0.0030)
Lev	0.1187 ***	−0.0811	−0.0548
	(0.0034)	(0.1958)	(0.0556)
Roa	0.2657 ***	16.9600 ***	6.5119 ***
	(0.0132)	(0.7407)	(0.2104)
Size	−0.0221 **	1.7020 ***	−0.0236
	(0.0108)	(0.6000)	(0.1705)
Q	−0.0067 **	−0.3518 **	−0.0408
	(0.0032)	(0.1771)	(0.0503)
Cr	−0.0002	0.0281	0.0376 ***
	(0.0004)	(0.0200)	(0.0057)
Cash	0.0004 ***	−0.0982 ***	−0.0190 ***
	(0.0001)	(0.0060)	(0.0017)
Age	−0.0001	−0.0035 *	−0.0019 ***
	(0.0000)	(0.0021)	(0.0006)
Lgdp	−0.0064 ***	0.6135 ***	0.1272 ***
	(0.0009)	(0.0475)	(0.0135)
Lpeo	−0.0151 ***	0.4693 ***	0.1763 ***
	(0.0014)	(0.0772)	(0.0219)
Constant	−0.6290 ***	−49.7578 ***	−20.4601 ***
	(0.0429)	(2.4026)	(0.6802)
Observations	14,764	14,762	14,762
R-squared	0.2504	0.6833	0.4329
year FE	YES	YES	YES
individual FE	YES	YES	YES

Notes: ***, ** and * denote significance levels of 1%, 5% and 10%, respectively; values in brackets are robust standard errors of clustering, and “YES” indicates controlling for firm and year fixed effects, as below.

**Table 8:** Inspection by Central Environmental Inspection.

	(1)	(2)
Variable Name	Lnpa	Lnino
Gfinance	0.3546 ***	0.0415
	(0.0659)	(0.0509)
Lev	1.4120 ***	0.1384 ***
	(0.1900)	(0.0467)
Roa	14.1907 ***	0.1023 **
	(0.7455)	(0.0471)
Size	3.0946 ***	0.2609
	(0.6596)	(0.1859)
Q	−0.3629 **	−0.0415
	(0.1806)	(0.0509)
Cr	0.0122	0.0345 ***
	(0.0205)	(0.0058)
Cash	−0.3546 ***	−0.0712 ***
	(0.0659)	(0.0186)
Age	−0.0007	−0.0013 **
	(0.0022)	(0.0006)
Lgdp	0.6977 ***	0.1458 ***
	(0.0482)	(0.0136)
Lpeo	0.6315 ***	0.2116 ***
	(0.0785)	(0.0221)
Constant	49.4537 ***	20.3834 ***
	(2.4277)	(0.6841)
Observations	14,762	14,762
R-squared	0.3860	0.2861
year FE	YES	YES
individual FE	YES	YES

Notes: *** and ** denote significance levels of 1% and 5% respectively; values in brackets are robust standard errors of clustering, and “YES” indicates controlling for firm and year fixed effects, as below.

**Table 9:** Test for heterogeneity of test area categories.

	Eastern Region		Midwest	
Variable Name	Lnpa	Lnino	Lnpa	Lnino
Gfinance	0.2544 ***	0.1199 ***	0.2878	0.1037 ***
	(0.0760)	(0.0291)	(0.2220)	(0.0203)
Lev	−1.5318 ***	−0.4293 ***	−1.1666 ***	−0.2068 **
	(0.2427)	(0.0692)	(0.3049)	(0.0835)
Roa	15.9399 ***	6.9418 ***	9.9378 ***	3.6242 ***
	(0.9189)	(0.2621)	(1.2716)	(0.3484)
Size	3.4461 ***	0.3492	2.4501 **	0.1290
	(0.8289)	(0.2364)	(1.0948)	(0.3000)
Q	−0.1978	0.0234	−0.5191 **	−0.1101
	(0.2506)	(0.0715)	(0.2575)	(0.0706)
Cr	0.0424 *	0.0440 ***	−0.0594 *	0.0099
	(0.0255)	(0.0073)	(0.0341)	(0.0093)
Cash	−0.4684 ***	0.2181 ***	−0.2510 ***	−0.0330
	(0.1019)	(0.0623)	(0.0866)	(0.0237)
Age	−0.0037	−0.0027 ***	0.0042	0.0012
	(0.0027)	(0.0008)	(0.0036)	(0.0010)
Lgdp	0.4893 ***	0.1037 ***	1.0691 ***	0.2228 ***
	(0.0712)	(0.0203)	(0.0799)	(0.0219)
Lpeo	0.8748 ***	0.2470 ***	−0.6511 ***	−0.0371
	(0.1719)	(0.0490)	(0.2437)	(0.0668)
Constant	−56.5180 ***	−23.7593 ***	−25.1902 ***	−11.2769 ***
	(3.4385)	(0.9806)	(4.5248)	(1.2399)
Observations	9812	9812	4950	4950
R-squared	0.5620	0.5109	0.4518	0.2107
year FE	YES	YES	YES	YES
individual FE	YES	YES	YES	YES

Notes: ***, ** and * denote significance levels of 1%, 5% and 10%, respectively; values in brackets are robust standard errors of clustering, and “YES” indicates controlling for firm and year fixed effects, as below.

**Table 10:** Regression results for enterprises with different ownership types.

	State-Owned Enterprises		Non-State Enterprises	
Variable Name	Lnpa	Lnino	Lnpa	Lnino
Gfinance	0.3144 ***	0.1366 ***	0.2448 ***	0.0681
	(0.0582)	(0.0492)	(0.0311)	(0.0762)
Lev	−1.6134 ***	−0.5165 ***	−1.1092 ***	−0.2141 ***
	(0.3492)	(0.0992)	(0.2336)	(0.0657)
Roa	19.8432 ***	6.9856 ***	13.0545 ***	5.4431 ***
	(1.2581)	(0.3573)	(1.0155)	(0.2855)
Size	2.1263 *	−0.3477	2.6512 ***	0.3779
	(1.2698)	(0.3607)	(0.9295)	(0.2614)
Q	−0.4246 *	−0.0528	−0.0356	0.0177
	(0.2231)	(0.0634)	(0.3728)	(0.1048)
Cr	0.1381 **	0.0608 ***	−0.0372 *	0.0250 ***
	(0.0633)	(0.0140)	(0.0224)	(0.0063)
Cash	−1.0033 ***	−0.1710 ***	−0.2501 ***	−0.0595 ***
	(0.2095)	(0.0595)	(0.0669)	(0.0188)
Age	−0.0070 *	−0.0012	0.0067 **	−0.0009
	(0.0040)	(0.0011)	(0.0026)	(0.0007)
Lgdp	0.8534 ***	0.2335 ***	0.4738 ***	0.0796 ***
	(0.0886)	(0.0252)	(0.0579)	(0.0163)
Lpeo	0.3121 **	0.1958 ***	0.7264 ***	0.2158 ***
	(0.1331)	(0.0378)	(0.1002)	(0.0282)
Constant	−64.2402 ***	−23.8839 ***	−46.1094 ***	−18.7830 ***
	(3.9420)	(1.1196)	(3.3214)	(0.9340)
Observations	5116	5116	9646	9646
R-squared	0.2590	0.1226	0.4950	0.6761
year FE	YES	YES	YES	YES
individual FE	YES	YES	YES	YES

Notes: ***, ** and * denote significance levels of 1%, 5% and 10%, respectively; values in brackets are robust standard errors of clustering, and “YES” indicates controlling for firm and year fixed effects, as below.

**Table 11:** Tests for industry heterogeneity of firms.

	Heavy Polluting Industries		Non-Heavily Polluting Industries	
Variable Name	Lnpa	Lnino	Lnpa	Lnino
Gfinance	0.1381 **	0.0738	0.2562 ***	0.1940 **
	(0.0633)	(0.0564)	(0.0895)	(0.0779)
Lev	−1.5549 ***	−0.4125 ***	19.5843 ***	7.4781 ***
	(0.2329)	(0.0644)	(1.2197)	(0.3510)
Roa	10.8162 ***	4.8587 ***	2.9866 ***	−0.0350
	(0.9144)	(0.2529)	(1.0683)	(0.3074)
Size	3.0662 ***	0.4337 *	−0.1383	0.0690
	(0.8167)	(0.2258)	(0.2870)	(0.0826)
Q	−0.5999 ***	−0.1357 **	−0.0092	0.0259 ***
	(0.2249)	(0.0622)	(0.0313)	(0.0090)
Cr	0.0222	0.0409 ***	−0.3281 ***	−0.0690 ***
	(0.0267)	(0.0074)	(0.0926)	(0.0266)
Cash	−0.3738 ***	−0.0688 ***	−0.0074 **	−0.0038 ***
	(0.0949)	(0.0262)	(0.0034)	(0.0010)
Age	0.0089 ***	0.0018 **	0.5264 ***	0.1484 ***
	(0.0027)	(0.0007)	(0.0824)	(0.0237)
Lgdp	0.7294 ***	0.1349 ***	0.6299 ***	0.2550 ***
	(0.0579)	(0.0160)	(0.1287)	(0.0370)
Lpeo	0.5215 ***	0.1633 ***	0.1601	0.1389 *
	(0.0962)	(0.0266)	(0.2812)	(0.0809)
Constant	−38.7261 ***	−16.7618 ***	−64.5687 ***	−25.5760 ***
	(2.9724)	(0.8220)	(3.9895)	(1.1479)
Observations	7910	7910	6852	6852
R-squared	0.5862	0.6477	0.6621	0.1047
year FE	YES	YES	YES	YES
individual FE	YES	YES	YES	YES

Notes: ***, ** and * denote significance levels of 1%, 5% and 10%, respectively; values in brackets are robust standard errors of clustering, and “YES” indicates controlling for firm and year fixed effects, as below.

## Data Availability

Not applicable.
